# Adherence to MHRA Guidelines for Valproate Prescribing in General Adult Psychiatry

**DOI:** 10.1192/bjo.2025.10586

**Published:** 2025-06-20

**Authors:** Muhammad Talha Farooq

**Affiliations:** Gartnavel Royal Hospital, Glasgow, United Kingdom

## Abstract

**Aims:** Valproate is widely prescribed for psychiatric conditions, particularly bipolar disorder, but carries significant teratogenic risks. The Medicines and Healthcare products Regulatory Agency (MHRA) mandates strict prescribing guidelines, particularly for women of childbearing potential, to mitigate these risks. Additionally, emerging evidence suggests potential risks for men, including infertility and neurodevelopmental concerns.

This audit aimed to assess adherence to MHRA guidelines within a Community Mental Health Team (CMHT), focusing on the documentation of risk discussions for all patients under 55 prescribed valproate, including discussions on infertility risks for men. It also evaluated the completion of risk acknowledgement forms for eligible patients and the enrolment of women of childbearing potential in the Pregnancy Prevention Programme (PPP). A secondary aim was to implement a targeted intervention and reassess compliance in a second audit cycle.

**Methods:** A retrospective review of electronic patient records was conducted for all patients under 55 prescribed valproate for psychiatric conditions at the CMHT. Patients with neurology-led prescriptions or aged over 55 were excluded. The first cycle, conducted in August 2024, included 22 patients (16 male, 6 female). Compliance with MHRA standards was assessed based on documented discussions, risk acknowledgement forms, and PPP enrolment. Following the first cycle, an intervention was introduced in the form of an email sent out to prescribers, emphasizing guideline adherence and areas for improvement. A second audit cycle was conducted in December 2024 to evaluate the impact of this intervention.

**Results:** The first audit cycle identified suboptimal compliance, particularly for male patients. Risk discussions were documented for all 6 female patients (100%) but only for 7 out of 16 male patients (43.75%). Risk acknowledgement forms were completed for 4 out of 6 female patients (66.67%). PPP enrolment was achieved in 3 out of 5 eligible female patients (60%).Following the email intervention, the second cycle demonstrated improvements. Risk discussions were documented for 9 out of 16 male patients (56.25%). Completion of risk acknowledgement forms improved to 5 out of 6 female patients (83.33%). PPP enrolment increased to 4 out of 5 eligible female patients (80%).

**Conclusion:** This audit highlights gaps in adherence to MHRA guidelines, particularly in documenting risk discussions for both male and female patients. The email intervention effectively improved compliance, but further efforts are needed. Future recommendations include electronic reminders in health records, and ongoing audits to ensure sustained adherence. Continuous clinician education is essential to enhance patient safety and regulatory compliance in valproate prescribing.

